# Predicting a low cortisol response to adrenocorticotrophic hormone in the critically ill: a retrospective cohort study

**DOI:** 10.1186/cc5928

**Published:** 2007-05-24

**Authors:** Margriet FC de Jong, Albertus Beishuizen, Jan-Jaap Spijkstra, Armand RJ Girbes, Rob JM Strack van Schijndel, Jos WR Twisk, AB Johan Groeneveld

**Affiliations:** 1 Department of Intensive Care, Institute for Cardiovascular Research, Vrije Universiteit Medical Center, De Boelelaan, 1081 HV Amsterdam, The Netherlands; 2 Department of Epidemiology and Biostatistics, Institute for Cardiovascular Research, Vrije Universiteit Medical Center, De Boelelaan, 1081 HV Amsterdam, The Netherlands

## Abstract

**Introduction:**

Identification of risk factors for diminished cortisol response to adrenocorticotrophic hormone (ACTH) in the critically ill could facilitate recognition of relative adrenal insufficiency in these patients. Therefore, we studied predictors of a low cortisol response to ACTH.

**Methods:**

A retrospective cohort study was conducted in a general intensive care unit of a university hospital over a three year period. The study included 405 critically ill patients, who underwent a 250 μg ACTH stimulation test because of prolonged hypotension or need for vasopressor/inotropic therapy. Plasma cortisol was measured before and 30 and 60 min after ACTH injection. A low adrenal response was defined as an increase in cortisol of less than 250 nmol/l or a peak cortisol level below 500 nmol/l. Various clinical variables were collected at admission and on the test day.

**Results:**

A low ACTH response occurred in 63% of patients. Predictors, in multivariate analysis, included sepsis at admission, low platelets, low pH and bicarbonate, low albumin levels, high Sequential Organ Failure Assessment score and absence of prior cardiac surgery, and these predictors were independent of baseline cortisol and intubation with etomidate. Baseline cortisol/albumin ratios, as an index of free cortisol, were directly related and increases in cortisol/albumin were inversely related to disease severity indicators such as the Simplified Acute Physiology Score II and Sequential Organ Failure Assessment score (Spearman *r *= -0.21; *P *< 0.0001).

**Conclusion:**

In critically ill patients, low pH/bicarbonate and platelet count, greater severity of disease and organ failure are predictors of a low adrenocortical response to ACTH, independent of baseline cortisol values and cortisol binding capacity in blood. These findings may help to delineate relative adrenal insufficiency and suggest that adrenocortical suppression occurs as a result of metabolic acidosis and coagulation disturbances.

## Introduction

Acute and severe illness is accompanied by increased serum levels of adrenocorticotrophic hormone (ACTH) and cortisol [1-21]. Even elevated levels may be too low for the level of physiological stress and may be associated with diminished adrenal responsiveness to additional stress, so-called relative adrenal insufficiency (RAI). The most commonly used test to assess adrenal function is the short ACTH stimulation test, in which serum cortisol is measured at baseline and up to 60 min after intravenous administration of 250 μg of synthetic ACTH [2-4,6-12,14,15,17-21]. A wide range exists in the prevalence of RAI among critically ill patients, varying from 0% to 77% [1-15,17-20,22,23]. This is partly due to the heterogeneity of case-mix and of criteria for presumably insufficient cortisol response to ACTH, although a low response is most commonly empirically defined as an increase of less than 250 nmol/l (9 μg/dl) [4,8-11,15,17-19,22].

Although there are no specific signs and symptoms of absolute adrenal insufficiency, several factors may be associated with RAI [5,7-10,12,13,15,22-24]. Remarkably, the literature is scarce and highly controversial on predictors and manifestations of RAI. Factors that are potentially associated with a low adrenal response are the presence of sepsis and shock [2,4-9,11-13,15,16,19,22,24], high lactate [10], hypoalbuminaemia [14,19], use of etomidate for intubation, mechanical ventilation and a low arterial oxygen tension/fractional inspired oxygen ratio [7,16,20,22,24,25], antifungal agents [26], high percentage of eosinophils [8,12,13,24], low sodium and glucose [12,13], and severe underlying disease or organ failure [7,9,10,16,22,23]. However, it is unknown whether these factors are interdependent [22]. In addition, low albumin and cortisol-binding globulin (CBG) levels may lower binding capacity in blood, and this may decrease total but maintain free cortisol levels. Hence, total cortisol level may be a poor indicator of whether adrenal cortisol secretion is adequate for the degree of physiologic stress exhibited by critically ill patients [6,14,16,19,27-29]. Indeed, although ACTH has no effect on albumin or CBG levels, the rise in total cortisol may be lower for a given rise in free cortisol when binding capacity is low [14,19,28]. Delineation of predictors and characteristics of RAI may help the clinician to select patients for ACTH testing. This may be important, because the results of the ACTH test may help to guide therapy with corticosteroids and thereby improve outcomes, particularly in vasopressor-refractory septic shock, although this is contoversial [6,8,9,11,13,17].

With the aim being to enhance understanding of RAI, the present study was undertaken to evaluate predictors of a low ACTH-induced cortisol response (exhibited by the so-called low responders), taking into account the severity of illness, baseline cortisol levels and hypoalbuminaemia. Therefore, a retrospective cohort study was conducted in 405 critically ill patients in whom an ACTH test was performed during the course of disease in our intensive care unit (ICU). The results of this analysis suggest that low pH/bicarbonate and low platelets, and greater severity of disease and organ failure are predictors of a subnormal increase in serum cortisol upon ACTH stimulation in a large series of critically ill patients; furthermore, these predictors were independent of sepsis, baseline cortisol and cortisol binding.

## Materials and methods

### Study population and adrenocorticotrophic hormone test

The present retrospective cohort study was conducted in the ICU of a teaching hospital (VU University Medical Center, Amsterdam, The Netherlands) over a period of three years. The study retrospectively included all patients admitted during this period who underwent a short ACTH (tetracosactide-hexa-acetate; Synacthen^®^; Novartis Pharma, Basel, Switzerland) stimulation test and for whom cortisol levels at baseline and 30 and 60 min after administration of 250 μg ACTH intravenously were available. The need for informed consent was waived because the test was performed on clinical and not investigational grounds. The Dutch legislation does not require informed consent for retrospective studies, provided that the results are anonymous. The test was performed in any patient who was suspected of having some degree of adrenocortical dysfunction on the basis of prolonged hypotension (> 6 hours requiring repeated fluid challenges) or need for vasopressors or inotropic drugs. Blood samples for serum cortisol measurement were taken immediately before (t = 0), and 30 min (t = 30) and 60 min (t = 60) after intravenous injection of ACTH. Serum cortisol was measured by competitive immunoassay (ASC-180 System; Bayer Diagnostics, Mijdrecht, The Netherlands). The coefficients of variation for this measurement are 3% for intra-assay variation and 6% for the interassay variation, and the detection limit is 30 nmol/l (500 nmol/l = 18 μg/dl). Whether treatment with corticosteroids was initiated after the test was at the discretion of the intensivists.

### Data collection

On the day of admission, general characteristics including age, sex, type of admission and underlying disease were recorded. International Classification of Disease-10 definitions were used for common clinical conditions at admission. The severity of illness was assessed by calculating the Simplified Acute Physiology Score (SAPS) II (range 0 to 163) and its associated predicted hospital mortality [30] and the Sequential Organ Failure Assessment (SOFA) score (range 0 to 24) [31], both at admission and on the day of the ACTH test, including haemodynamic, pulmonary, renal, neurological, infectious and biochemical parameters. Multiple organ dysfunction was defined as a SOFA score of 7 or greater. The worst values within a 24 hour period were used to calculate the scores. Missing values were regarded as normal. Sepsis at the ACTH test day was defined as the presence of systemic inflammatory response syndrome with a positive microbiological local (trachea, urine, or other) or blood culture, or both. Systemic inflammatory response syndrome was defined was a temperature above 38°C or below 35.5°C, a leucocyte count above 12 × 10^9^/l or below 4 × 10^9^/l, a heart rate above 90 beats/min, and a respiratory rate above 20 breaths/min or need for mechanical ventilation.

Prior use of drugs that may interfere with adrenocortical function, including corticosteroids and antifungal agents [26], from one month before until the test day was reported, as well as the day of intubation. Etomidate is often used to facilitate intubation in our institution. Interventions such as type and dose of inotropics, treatment with corticosteroids, mechanical ventilation and renal replacement therapy were reported, as were positive cultures of trachea, urine, blood and other local sites of infection from seven days before to the day of the ACTH test. The Glasgow Coma Scale (GCS) score recorded was the GCS before sedation in patients on sedatives.

A low response to ACTH in critical illness (RAI) was defined as a cortisol increase of less than 250 nmol/l [4,8-11,15,17-19,22] or a peak level below 500 nmol/l [2,4,5,14,21]. To estimate free cortisol at baseline and its increase following ACTH stimulation, values were normalized for serum albumin (cortisol/albumin ratio) when available (*n *= 332). Mortality was defined as death in the ICU until day 28 after admission, or as hospital mortality.

### Statistical analysis

We conducted a Fisher's exact test for categorical variables and a Mann-Whitney *U*-test for continuous variables (SPSS version 11; SPSS Inc., Chicago, IL, USA). All variables differing among groups at a *P *< 0.10 level and available for at least 95% (model 1) or 75% or more (model 2) of the patients were entered into a backward stepwise multiple logistic regression model with low ACTH response (either a low increase [model a] or peak [model b]) as the dependent variable. Hence, models 1a and 1b (for low increase and peak, respectively) did not include albumin levels, whereas models 2a and 2b did. When variables were recorded both on admission and on the test day, such as SAPS II and SOFA scores, variables recorded on the test day were included only, and either the presence of sepsis on admission or on the test day was considered. The Hosmer-Lemeshow test was used to evaluate the goodness-of-fit. Odds ratios (95% confidence intervals) were calculated for categorical data. Final prediction models were validated using a bootstrap method for 1,000 replicates (Stat version 9; StataCorp LP, College Station, TX, USA). We identified the maximum number of replicates (validity) as 100% minus the minimum percentage (at 5%, 10%, 20%, 50%, 80%, 90% and 95%) of replicates to achieve statistical significance for each predictor. The Kruskal-Wallis test was used to compare baseline cortisol levels and increases, normalized for albumin levels, in predefined strata of SAPS II and SOFA scores. Data are expressed as median (range). A two-sided *P *< 0.05 was considered to indicate statistical significance, and exact *P *values are given unless they are less than 0.0001.

## Results

### Patient characteristics

In all, 405 patients were included. Age and sex distribution and mortality rate among the study population and all other patients (*n *= 3,953) admitted to our ICU during the study period did not differ. However, fewer patients in the study population were admitted after trauma and surgery (*P *< 0.0001) and more were admitted after heart surgery or cardiopulmonary resuscitation, with respiratory failure, shock, renal failure (*P *< 0.0001), or sepsis (*P *= 0.002). Table 1 shows the clinical characteristics of responders and low responders (58% for an increase in cortisol < 250 nmol/l, 32% for peak cortisol <500 nmol/l, and 63% for either). Low responders were more often admitted with sepsis, and their admission SAPS II and SOFA scores were higher than in responders. Accordingly, mortality was higher in low responders with an increase below 250 nmol/l, although they were more likely to have received corticosteroids.

**Table 1:** Patient characteristics according to cortisol response

Characteristic	Increase ≥250 nmol/l (*n *= 170)	Increase <250 nmol/l (*n *= 235)	*P*	OR (95% CI)	Peak ≥500 nmol/l (*n *= 276)	Peak <500 nmol/l (*n *= 129)	*P*	OR (95% CI)
Age (years)	63 (17–88)	65 (15–93)			65 (17–93)	61 (15–89)		
Sex (male/female)	110 (65)/60 (35)	151 (64)/84 (36)			169 (61)/107 (39)	92 (71)/37 (29)	0.058	0.64 (0.40–1.00)
Underlying disease								
Cardiovascular	92 (54)	99 (42)	0.020	0.62 (0.42–0.92)	138 (50)	53 (41)		
Renal	2 (1)	6 (3)			4 (1)	4 (3)		
Pulmonary	10 (6)	20 (9)			21 (8)	9 (7)		
Hepatic	1 (1)	12 (5)	0.010	9.09 (1.17–70.63)	7 (3)	6 (5)		
Gastrointestinal	11 (6)	26 (11)			19 (7)	18 (14)	0.026	2.19 (1.11–4.33)
Neurological	13 (8)	15 (6)			20 (7)	8 (6)		
Endocrinological	18 (11)	27 (11)			35 (13)	10 (8)		
Cancer	19 (11)	32 (14)			35 (13)	16 (12)		
Admission syndromes^a^								
Trauma and post-operative	79 (46)	92 (39)			114 (43)	57 (44)		
Cardiac surgery	39 (23)	26 (11)	0.002	0.42 (0.24–0.72)	47 (17)	18 (14)		
Vascular surgery	8 (5)	17 (7)			17 (6)	8 (6)		
Respiratory failure	47 (28)	72 (31)			85 (31)	34 (26)		
Post-CPR	14 (8)	10 (4)			20 (7)	4 (3)		
Sepsis	12 (7)	45 (19)	<0.0001	3.12 (1.59–6.10)	31 (11)	26 (20)	0.021	2.00 (1.13–3.52)
Shock	5 (3)	16 (7)			16 (6)	5 (4)		
Renal insufficiency	6 (4)	11 (5)			9 (3)	8 (6)		
Coma	3 (2)	8 (3)			8 (3)	3 (2)		
Other	35 (21)	58 (25)			49 (18)	26 (20)		
Admission SAPS II	36 (0–95)	44 (9–94)	<0.0001		39 (7–95)	42 (0–94)		
Admission SOFA	8 (0–17)	9 (0–22)	0.001		8 (0–18)	9 (0–22)	0.018	
CS after test	102 (60)	185 (79)	<0.0001	2.47 (1.59–3.82)	181 (66)	106 (82)	0.001	2.42 (1.45–4.05)
ICU mortality	24 (14)	63 (27)	0.002	0.45 (0.27–0.75)	54 (20)	33 (26)		
In CS-treated patients	16 (16)	48 (26)	0.054	0.53 (0.28–0.99)	37 (13)	27 (21)		
In non-CS-treated patients	8 (12)	15 (30)	0.018	0.31 (0.12–0.81)	17 (6)	6 (5)		
Hospital mortality	48 (28)	109 (46)	<0.0001	0.45 (0.30–0.69)	103 (37)	54 (42)		

Values are expressed as median (range) or number (%), where appropriate. Exact *P *values are given where *P *< 0.10. ^a^Patients may have more than one condition. All variables were scored for 100% of the patients. 500 nmol/l = 18 μg/dl cortisol. CI, confidence interval; CPR, cardiopulmonary resuscitation; CS, corticosteroids; ICU, intensive care unit; SAPS, Simplified Acute Physiology Score; SOFA, Sequential Organ Failure Assessment.

### Adrenocorticotrophic hormone test

For the entire population, median (range) baseline cortisol was 360 nmol/l (30–1,870 nmol/l), the median cortisol increase was 210 nmol/l (-180 to +1,015 nmol/l), and median peak cortisol was 610 nmol/l (30 to 1,950 nmol/l). Table 2 describes lower cortisol/albumin ratios in low responders, among others.

**Table 2:** Results of the adrenocorticotrophic hormone test

Parameter	Increase ≥250 nmol/l (*n *= 170)	Increase <250 nmol/l (*n *= 235)	*P*	Peak ≥500 nmol/l (*n *= 276)	Peak <500 nmol/l (*n *= 129)	*P*
Baseline cortisol (nmol/l)	323 (40–1160)	375 (30–1870)	0.014	435 (49–1870)	220 (30–475)	< 0.0001
Baseline cortisol/albumin (nmol/g)	21.6 (2–66)	29.0 (1–198)	< 0.0001	28.6 (2–198)	16.5 (1–62)	< 0.0001
t = 30 cortisol (nmol/l)	635 (255–1740)	475 (30–1910)	< 0.0001	640 (290–1910)	350 (30–485)	< 0.0001
t = 60 cortisol (nmol/l)	710 (335–1720)	510 (30–1950)	< 0.0001	690 (350–1950)	375 (30–495)	< 0.0001
Peak cortisol (nmol/l)	710 (335–1740)	520 (30–1950)	< 0.0001	695 (500–1950)	385 (30–495)	< 0.0001
Peak <500 nmol/l	20 (12)	109 (46)	< 0.0001	na	na	
Cortisol increase (nmol/l)	358 (250–1015)	130 (-180–245)	< 0.0001	268 (-180–1015)	135 (-100–373)	< 0.0001
Cortisol increase/albumin (nmol/g)	21.7 (8–54)	8.0 (-8–43)	< 0.0001	17.1 (-8–54)	9.7 (-8–43)	< 0.0001
Cortisol increase <250 nmol/l	na	na	126 (46)	109 (84)	< 0.0001	

Values are expressed as median (range) or number (%), where appropriate. Exact *P *values are given where *P *< 0.10 500 nmol/l = 18 μg/dl cortisol. na, not applicable.

### Predictors

Table 3 describes statistically significant clinical and biochemical predictors of a diminished ACTH response, as identified in univariate analysis. Availability of data is indicated. Of the 57 patients with sepsis at admission 39 had sepsis on the ACTH test day, whereas 179 additional patients fulfilled sepsis criteria on the ACTH test day (*P *= 0.021). Disease severity was greater in low responders. Heart rate was higher in low responders (cortisol increase < 250 nmol/l) and dependency on vasopressor therapy was greater, and they more frequently received ventilatory support at higher fractional inspired oxygen. Of all patients, 96% were intubated, and intubation was significantly associated with a low response (peak cortisol < 500 nmol/l). Low responders (cortisol increase < 250 nmol/l) also had a shorter interval between admission/intubation and the ACTH test than did responders. In low responders (cortisol increase < 250 nmol/l), lower urinary production was accompanied by higher serum creatinine and urea levels. The pH and bicarbonate concentrations were lower in low responders. Furthermore, they had lower platelet counts and albumin levels, and those with a cortisol increase below 250 nmol/l also had lower glucose and a lower percentage of eosinophils in blood smears.

**Table 3:** Predictors of a low response to adrenocorticotrophic hormone

Factor	Increase ≥250 nmol/l (*n *= 170)	Increase <250 nmol/l (*n *= 235)	*P*	OR (95% CI)	Peak ≥500 nmol/l (*n *= 276)	Peak <500 nmol/l (*n *= 129)	*P*	OR (95% CI)
Time from admission (days)^a^	5 (1–77)	3 (1–92)	<0.0001		4 (1–77)	4 (1–92)		
Intubation^a^	161 (95)	229 (97)			261 (95)	129 (100)	0.004	na
Time until test (days)^a^	4 (0–76)	2 (0–70)	0.001		3 (0–76)	3 (0–70)		
Fluconazole^a^	12 (7)	29 (12)	0.077	1.85 (0.92–3.75)	22 (8)	19 (15)	0.050	2.00 (1.04–3.83)
Time until test (days)	4 (0–15)	7 (0–63)	0.038		6 (0–63)	5 (0–53)		
SAPS II*	35 (7–97)	44 (7–100)	<0.0001		39 (9–97)	40 (7–100)		
SOFA*	8 (0–21)	10 (0–21)	<0.0001		8 (0–21)	10 (0–21)	0.014	
Multiple organ dysfunction^a^	103 (61)	194 (83)	<0.0001	3.08 (1.95–4.86)	192 (70)	105 (81)	0.016	1.92 (1.15–3.19)
Heart rate (beats/min)^a^	90 (48–146)	96 (59–171)	0.001		94 (48–171)	94 (52–146)		
Vasopressors/inotropes^a^	134 (79)	206 (88)	0.020	1.91 (1.12–3.26)	226 (82)	114 (88)		
Mechanical ventilation^a^	152 (89)	224 (95)	0.031	2.41 (1.11–5.25)	250 (91)	126 (98)	0.012	4.37 (1.30–14.71)
FiO_2_^b^	0.41 (0.29–1.0)	0.50 (0.34–1.0)	<0.0001		0.45 (0.29–1.0)	0.49 (0.30–1.0)		
PaO_2_/FiO_2_^b^	240 (59–681)	203 (44–641)	0.005		220 (44–681)	225 (77–641)		
Renal replacement^a^	23 (14)	47 (20)			35 (13)	35 (27)	0.001	2.56 (1.52–4.33)
Urine production (ml)^a^	255 (0–8845)	1667 (0–6970)	<0.0001		1989 (0–10140)	1697 (0–6970)		
Creatinine (μmol/l)^a^	92 (23–695)	122 (20–1934)	<0.0001		112 (20–1934)	116 (36–675)		
Urea (mmol/l)^b^	10.2 (0.7–46.3)	13.3 (1.5–149)	0.018		12.1 (1.0–149)	12.4 (0.7–89.8)		
Glasgow Coma Scale score^a^	15 (3–15)	11 (3–15)	0.070		11 (3–15)	15 (3–15)		
Positive other local culture^a^	50 (29)	88 (37)			76 (28)	62 (48)	<0.0001	2.43 (1.58–3.76)
SIRS^a^	116 (68)	194 (83)	0.001	2.20 (1.38–3.51)	213 (77)	97 (75)		
Sepsis^a^	80 (47)	138 (59)	0.021	1.60 (1.08–2.38)	147 (53)	71 (55)		
Haemoglobin (mmol/l)^a^	5.8 (4.0–7.9)	5.7 (3.6–9.7)			5.8 (4.0–8.8)	5.6 (3.6–9.7)	0.011	
Haematocrit^a^	0.28 (0.20–0.38)	0.27 (0.17–0.44)			0.28 (0.20–0.43)	0.26 (0.17–0.44)	0.004	
Platelets (× 10^9^/l)^a^	192 (20–818)	130 (3–756)	<0.0001		173 (4–818)	123 (3–468)	0.001	
Eosinophils (%)	1 (0–5)	0 (0–4)	0.014		0 (0–5)	0 (0–1)		
Albumin (g/l)^b^	17 (6–32)	13 (3–34)	<0.0001		17 (3–34)	12 (3–32)	<0.0001	
Bilirubin (μmol/l)^c^	10 (3–176)	14 (2–441)	0.001		12 (2–280)	12 (2–441)		
Arterial pH^a^	7.43 (7.07–7.56)	7.38 (6.89–7.64)	<0.0001		7.41 (6.89–7.64)	7.39 (7.02–7.54)	0.039	
Bicarbonate (mmol/l)^a^	25.0 (6.6–37.8)	21.7 (6.8–37.0)	<0.0001		23.8 (6.6–37.8)	21.9 (12.1–33.4)	0.005	
Glucose (mmol/l)^a^	7.6 (1.9–35.0)	7.1 (2.3–25.8)	0.036		7.3 (2.6–35.0)	7.1 (1.9–25.8)		

Values are expressed as median (range) or number (%), where appropriate. Exact *P *values are given where *P *< 0.10. ^a^Data available in >95%. ^b^Data available in ≥75%. ^c^Data available in 64%. 500 nmol/l = 18 μg/dl cortisol. CI, confidence interval; FiO_2_, inspired fractional oxygen; HR, heart rate; na, not applicable; PaO_2_, partial arterial oxygen tension; SAPS, Simplified Acute Physiology Score; SIRS, systemic inflammatory response syndrome; SOFA, Sequential Organ Failure Assessment.

### Correlations and multivariate analyses

**Figure 1:**
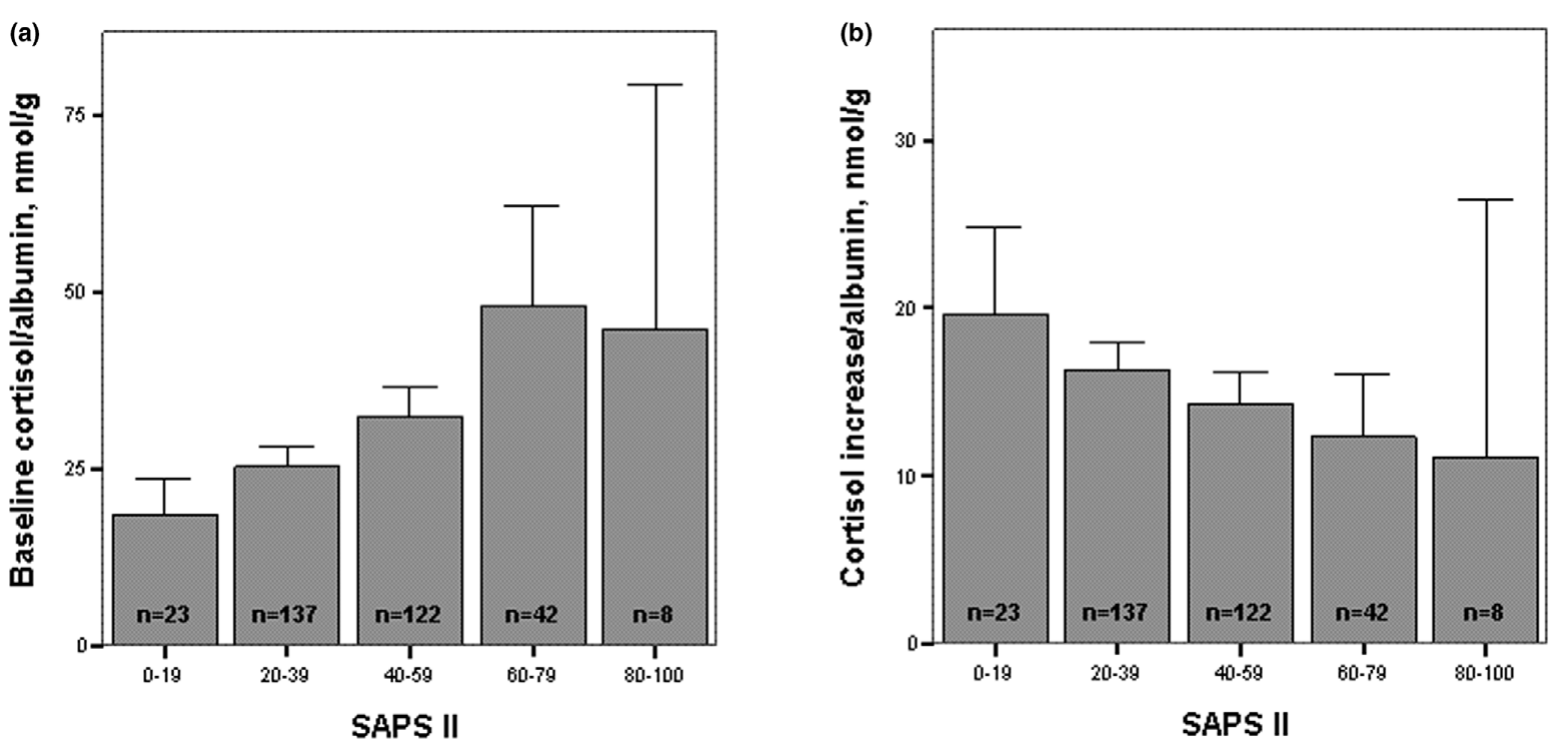
Relation between baseline and ACTH-induced increases in cortisol/albumin and SAPS II score. **(a) **Association between baseline cortisol/albumin and Simplified Acute Physiology Score (SAPS) II score (five strata; *P *< 0.0001, Kruskal-Wallis test). **(b) **Association between adrenocorticotrophic hormone (ACTH)-induced increases in cortisol/albumin and SAPS II strata (*P *= 0.002, Kruskal-Wallis test).

There was little relation between increases in cortisol and baseline values (Spearman *r *[*r*_s_] = -0.17; *P *= 0.001). Both baseline cortisol values and increases were somewhat related directly to albumin levels (minimum *r*_s _= 0.17; *P *= 0.002). Baseline and increases in cortisol levels were related directly and inversely to SAPS II (minimum *r*_s _= 0.25, *P *< 0.0001), respectively, and SOFA scores (minimum *r*_s _= 0.12; *P *= 0.015). Figure 1 shows the relation between strata of SAPS II scores and baseline and increases in the cortisol/albumin ratio (as an index of free cortisol), which suggests that a relation exists between severity of illness on the one hand and free cortisol and diminished rises in cortisol upon ACTH stimulation on the other hand (minimum *r*_s _= -0.22; *P *< 0.0001). Similarly, strata of SOFA scores exhibited direct and inverse relations with baseline cortisol/albumin ratios and ACTH-induced increases in cortisol/albumin (*P *= 0.003 and *P *= 0.001), respectively. Increases in cortisol and in cortisol/albumin ratio were related to platelet counts, pH and bicarbonate (*P *= 0.006 or lower).

Table 4 shows the results of multivariate analyses, using variables available in 95% or more (models 1a and 1b) or 75% or more (models 2a and 2b) of patients, conducted to identify factors that predict low increases or peaks. The results show that high SOFA score, low platelet count, low pH, and low bicarbonate and low albumin levels were, in descending order, the most frequent predictors of low response, and these predictors were independent of each other and baseline cortisol. In contrast, prior cardiac surgery protected. Modeling the data with inclusion of sepsis on the test day rather than at admission to predict a cortisol increase below 250 nmol/l yielded similar results for platelet count, pH, albumin and cardiac surgery, independently of SOFA, baseline cortisol, time from admission/intubation until test and use of etomidate.

**Table 4:** Predictors of a low adrenocortictrophic hormone response in multivariate analysis

Factor	Increase <250 nmol/l			Peak <500 nmol/l		
	
	OR (95% CI)	*P*	Validity		OR (95% CI)	*P*
	**Model 1a (*n *= 404)**			**Model 1b (*n *= 403)**		

Positive other local culture	na			2.40 (1.32–4.37)	0.004	<5%
Sepsis at admission	2.34 (1.13–4.84)	0.022	<5%	na		
SOFA test day	1.11 (1.03–1.18)	0.003	50–80%	1.11 (1.01–1.21)	0.024	50–80%
Baseline cortisol (nmol/l)	1.001 (1.000–1.002)	0.045	50–80%	0.987 (0.985–0.990)	<0.0001	>95%
Platelets (× 10^9^/l)	0.998 (0.996–1.000)	0.031	50–80%	0.997 (0.994–1.000)	0.044	50–80%
Bicarbonate (mmol/l)	na			0.91 (0.86–0.97)	0.003	80–90%
Cardiac surgery	0.48 (0.26–0.86)	0.013	90–95%	na		
Arterial pH	0.011 (0.001–0.185)	0.002	>95%	na		

	**Model 2a (*n *= 330)**			**Model 2b (*n *= 332)**		

SOFA test day	na			1.19 (1.09–1.30)	<0.0001	90–95%
Heart rate (beats/min)	1.015 (1.003–1.028)	0.015	50–80%	na		
Platelets (× 10^9^/l)	0.997 (0.995–0.999)	0.009	50–80%	na		
Baseline cortisol (nmol/l)	na			0.986 (0.983–0.990)	<0.0001	>95%
Albumin (g/l)	0.93 (0.90–0.97)	0.001	90–95%	0.92 (0.87–0.97)	0.003	80–90%
Cardiac surgery	0.40 (0.20–0.79)	0.008	>95%	na		
Arterial pH	0.002 (0.0001–0.048)	<0.0001	>95%	na		

Models 1 and 2 included all univariate significant variables that were available in at least 95% and 75% of patients, respectively (see text). Validity was assessed by bootstrap analysis (see Materials and methods). Hosmer-Lemeshow tests: model 1a: χ^2 ^= 9.2, degrees of freedom (df) = 8, *P *= 0.32; model 1b: χ^2 ^= 4.4, df = 8, *P *= 0.82; model 2a: χ^2 ^= 13.2, df = 8, *P *= 0.11; and model 2b: χ^2 ^= 10.0, df = 8, *P *= 0.26. CI, confidence interval; na, not applicable; OR, odds ratio; SOFA, Sequential Organ Failure Assessment.

## Discussion

The main finding of the present study, comprising the largest series of ACTH tests in general ICU patients thus far reported, is the value of a set of clinical parameters for predicting RAI during critical illness. The set consisted of low arterial pH, low bicarbonate, low platelet count and high SOFA score, particularly in noncardiac (surgical) patients, and these predictors were independent of sepsis, interval until testing, intubation with etomidate, baseline cortisol and albumin levels. The results not only help in predicting a diminished response to ACTH stimulation but also provide insight into the pathophysiological mechanisms of a low response and significance of RAI. That low pH/bicarbonate is predictive of RAI can be explained by underlying circulatory insufficiency and perhaps adrenal hypoperfusion, or by metabolic acidosis directly suppressing adrenal cortisol synthesis [32]. However, lactate levels did not differ among responders and low responders, thereby arguing against the former. The contribution of low platelets to a low response, independent of sepsis or infection, may be caused by circulating factors promoting platelet aggregation and impairing adrenal function; alternatively, it may be associated with adrenal microcirculatory thrombosis or bleeding, which are known to impair cortisol synthesis [33].

We used a cortisol increase of 250 nmol/l and a peak level of 500 nmol/l as the cutoff values to define RAI [2,4,5,8-11,14,15,17-19,21,22], even though our data indicate a continuum of baseline cortisol and increases in cortisol values rather than a bimodal distribution. We did not exclude patients with very low baseline cortisol values or increases, which are partly attributable to low protein binding during critical illness [14,19]; this contributes to poor differentiation between absolute adrenocortical dysfunction and RAI in these patients. Although widely varying definitions and cutoff values have been used, and corresponding prevalences of RAI greatly differ between studies, an increase of less than 250 nmol/l appears to be associated with the greatest predictive value for steroid responsiveness in septic shock and mortality, although this is controversial [6,8,9,11,13,17]. In any case, low increases can only partly be attributed to high baseline cortisol values, and the prevalence of RAI in the present study is in accordance with findings reported in the literature [6,7,9,10,13,15,22,23].

None of the classic signs and symptoms associated with adrenal insufficiency (for instance, fever, hyponatraemia and hyperkalaemia) was predictive of RAI in our patients, even though the blood glucose level was somewhat lower in low responders. Other investigators demonstrated an association of relative eosinophilia with low response to ACTH [8,12,13,24]. A lower percentage of eosinophils among low responders in our study could be attributed to somewhat higher baseline cortisol levels. In any case, advanced age was not a predictor, which is in accordance with many other reports [8,13,15,22]. Although prior cardiovascular disease or cardiac surgery was not associated with RAI, sepsis at admission, which was already present at admission in about 20% of low responders, was an independent predictor for a low response. This is in accordance with the literature, which indicates that there is a high incidence of RAI in patients with sepsis and shock [4,5,7-9,11-13,16,19,22,24]. Plasma from patients with septic shock impairs synthesis of corticosteroids by adrenocortical cells [34]. We evaluated predictors of low response in patients who had sepsis at admission and who met criteria for sepsis at the time of the ACTH test separately; these predictors appeared to be similar, in multivariate analyses. Because sepsis on the test day occurred in about 57% of low responders and was not an independent predictor, we cannot exclude the possibility that RAI also occurred in nonseptic hypotensive patients.

Low responders (cortisol increase < 250 nmol/l) were more often treated by vasopressors, which is in agreement with findings reported in the literature [5,7,8,10,15,22,24]. Etomidate is commonly used to facilitate endotracheal intubation; it is an inhibitor of 11β-hydroxylase, which is involved in cortisol synthesis. A single bolus of etomidate has been shown to diminish transiently the response to ACTH in critically ill patients [16,20,22,25]. Indeed, depression of adrenal function by etomidate may be transient, but lasts for at least 24 hours [20,22,25]. However, in our study, intubation with the help of etomidate and the interval between intubation and the test were associated with low response in univariate analysis but not in multivariate analysis. Mechanical ventilation did not predict a low ACTH response either, which is in contrast to the literature [17,24]. Similarly, prior treatment of fungal infection with fluconazole, which utilizes the cytochrome P450 system for metabolism and which inhibits 11β-hydroxylase, did not predict RAI in our study; this is in accordance with the literature [26].

This study has some limitations. By virtue of the study design and rationale, the patients studied represent a selected group. We did not separately score for head trauma in our patients, which may carry risk for endocrine dysfunction. Nevertheless, a GCS score below 8 in the presence of trauma did not contribute to prediction of a low cortisol response (peak or increase). The CBG and free cortisol levels were not directly measured, and we might have underestimated baseline free cortisol levels and rises upon ACTH stimulation, as pointed out previously [6,14,16,19,27-29]. However, we used albumin levels to estimate free cortisol, because albumin may also bind cortisol, albeit to a lesser extent than CBG, and both albumin and CBG levels may decrease to the same extent in critical illness [16,19,27-29]. Hamrahian and coworkers [14] also used blood albumin level as a surrogate marker of plasma cortisol binding capacity. Low albumin levels were associated with low baseline cortisol values and increases. However, hypoalbuminaemia independently increased the risk for a low response, suggesting that the latter was only partly caused by diminished cortisol-binding proteins; Ho and colleagues [19] concurred with this view, but Hamrahian and coworkers [14] attributed a low total cortisol response mainly to low serum cortisol-binding capacity. In groups divided on the basis of the Hamrahian criterion of an albumin level of less than (*n *= 309) or greater than 25 g/l (*n *= 23), there were no differences in baseline cortisol values and increases. Moreover, our findings with high baseline levels and low increases in cortisol associated with increasing severity of disease (Figure 1) and organ failure were not affected by cortisol binding, and the multivariate predictors of low responses were independent of baseline cortisol and albumin levels. Some studies [1-3], but not all, indeed suggest that (total) cortisol values increase and ACTH-induced increases diminish with increasing Acute Physiology and Chronic Health Evaluation II score or other disease severity and organ failure scores, unless limited by progressive and severe hypoalbuminaemia and decreased cortisol binding [1-3,7,10,19,21-23]. Hence, the diminished (total and free) cortisol response to ACTH was a marker of severity of disease in our critically ill patients. Nevertheless, we cannot determine whether the free cortisol response to ACTH was sufficient in terms of ability to cope with additional stress. Conversely, the existence of RAI is doubtful when baseline (free) cortisol levels are high or when 250 μg ACTH is regarded as a supraphysiologic stimulus [5,18,24].

## Conclusion

We conclude that low pH/bicarbonate and low platelets, and increased severity of disease and organ failure were predictors of a subnormal increase in serum cortisol upon ACTH stimulation in a large series of critically ill patients, and these predictors were independent of sepsis, baseline cortisol and cortisol binding. This suggests that adrenocortical suppression occurs as a result of metabolic acidosis and coagulation disturbances. Even though increases in cortisol form a continuum and the cutoff values chosen are relatively arbitrary, our findings may help to better define RAI, which may be associated with increased mortality.

## Key messages

• In a large series of critically ill patients, low pH/bicarbonate and low platelets, and increased severity of disease and organ failure were predictors of a subnormal increase in serum cortisol upon ACTH stimulation.

• These predictors are independent of sepsis, baseline cortisol and cortisol binding.

• Adrenocortical suppression may be caused, in part, by metabolic acidosis and coagulation disturbances.

## Abbreviations

ACTH = adrenocorticotrophic hormone; CBG = cortisol-binding globulin; GCS = Glasgow Coma Scale score; ICU = intensive care unit; RAI = relative adrenal insufficiency; SAPS = Simplified Acute Physiology Score; SOFA = Sequential Organ Failure Assessment.

## Competing interests

The authors declare that they have no competing interests.

## Authors' contributions

AB participated in the design of the study and helped to draft the manuscript. AG helped to draft the manuscript. JG participated in the design and coordination of the study, and helped to draft the manuscript. JS helped to draft the manuscript. JT participated in the statistical analysis. MJ participated in the design of the study, carried out the data collection, performed the statistical analysis, and drafted the manuscript. RS helped to draft the manuscript.

All authors read and approved the final manuscript.
